# A multianalyte method for simultaneous evaluation of occupational exposure to anesthetic gases in urine samples from healthcare professionals

**DOI:** 10.3389/fpubh.2026.1802993

**Published:** 2026-04-16

**Authors:** Lydia Espín-Moreno, Alfonso M. Durán-López, Ainhoa Pérez-Cantero, Alberto Benavente-Romero, Andrea Rodríguez-Carrillo, Vicente Mustieles, Tomás Saz-Terrado, Mariana F. Fernández

**Affiliations:** 1 Consortium for Biomedical Research in Epidemiology and Public Health (CIBERESP), Madrid, Spain; 2 Instituto de Investigación Biosanitaria (ibs.GRANADA), Granada, Spain; 3 Center for Biomedical Research (CIBM), University of Granada, Granada, Spain; 4 Anaesthesiology and Resuscitation Service, Hospital Universitario Clínico San Cecilio, Granada, Spain

**Keywords:** anesthetic gases, HS–GC–MS, method validation, non-invasive biomonitoring, occupational exposure, urine

## Abstract

**Background:**

Monitoring occupational exposure in hospital settings requires valid non-invasive exposure assessment methods. We aimed to develop a multianalyte method to characterize and quantify urinary levels of anesthetic gases in healthcare professionals.

**Methods:**

Urine samples were collected from 37 recently exposed and 24 non-exposed professionals. A multianalyte method was developed and validated to determine urinary concentrations of desflurane, isoflurane, sevoflurane, and its main metabolite, hexafluoroisopropanol, using headspace gas chromatography coupled to mass spectrometry (HS–GC–MS).

**Results:**

The methodology was successfully validated. It demonstrated adequate linearity and sensitivity [correlation coefficients (*R*^2^): 99.5–99.9%]. Limits of detection for the anesthetic gases ranged from 0.15–0.60 ng/mL, and limits of quantification from 0.5–2.0 ng/mL. The recovery percentage ranged from 95.17 to 102.40%, and relative standard deviations were below 15%. Detectable concentrations in exposed participants ranged from 1.0 to 3.80 ng/mL (mean 0.53 ng/mL) for desflurane, 0.5–19.59 ng/mL (mean 1.39 ng/mL) for sevoflurane, and 2.0–729.35 ng/mL (mean 49.74 ng/mL) for hexafluoroisopropanol (HFIP). No detectable concentrations of isoflurane were found in the exposed volunteers, nor were any of the anesthetic found in the non-exposed volunteers.

**Conclusion:**

A multianalyte HS–GC–MS method was successfully developed to simultaneously determine four anesthetic gases in urine samples and was validated in real-world samples from healthcare professionals. Future studies are needed to investigate the safety of exposure to these compounds for anesthesiologists and operating room nurses.

## 1 Introduction

Volatile anesthetics are extensively used in numerous surgical procedures. Despite advances in anesthetic equipment and improvements in the safety of anesthetic drugs, the occupational exposure of healthcare professionals and the potential risks to their health remain matters of concern (1). The most frequently utilized agents in general anesthesia are halogenated gases such as sevoflurane (SEV), desflurane (DES), isoflurane (ISO) and, to a lesser extent, nitrous oxide (N_2_O) (2). These initially liquid compounds are administered *via* closed or semi-open vaporization and breathing systems, allowing precise anesthetic control (3, 4).

SEV and DES have become the anesthetics of choice over recent decades due to their favorable pharmacokinetic profiles, i.e., their low blood solubility, rapid onset of action, quick emergence, and ready excretion from the central nervous system (5). DES is much stabler in comparison to SEV, with minimal degradation and metabolic transformation rates. However, the non-irritant nature of SEV facilitates a smoother respiratory transition during anesthesia induction, making it suitable for pediatric and cardiovascular procedures as well as for patients with bronchial disorders, given its bronchodilator and vasodilator effects (6–8). ISO is less frequently selected than SEV or DES because of its potential to irritate the respiratory tract and its longer recovery times (9). N_2_O, once a common choice, has declined in popularity due to concerns about its hematological side effects, environmental impact, potential neurotoxicity, and association with neurological complications and vitamin B12 deficiency (10, 11).

In general, the widespread use of these agents has raised concerns about waste anesthetic gases (WAGs), i.e., the small amounts of volatile anesthetic gases that leak from the patient’s breathing circuit into the air of operating rooms (2). Authors have associated exposure to WAGs with possible long-term genotoxic effects and oxidative stress in frequently exposed individuals such as healthcare professionals, and these effects may be especially relevant for women of childbearing age (12–14). Exposure to WAGs has been reported to increase the risk of chromosomal abnormalities and elevated micronucleus frequencies in the oral mucosa cells of anesthesiology interns (15). Relatively short-term exposure has been related to headaches, tiredness, and nausea, while long-term exposure has been associated with negative reproductive effects and genomic instability (12). In addition, researchers using different cytogenetic damage biomarkers described a consistent pattern of genetic impairment in healthcare professionals exposed to WAGs (15, 16). In relation to oxidative stress, decreased antioxidant capacity has been reported in healthcare workers exposed to WAGs, although study results have been inconsistent (17–19). Thus, some occupational studies associated WAG exposure with altered levels of oxidative stress biomarkers, including superoxide dismutase, glutathione peroxidase, and malondialdehyde (17), but another investigation found no significant differences with non-exposed controls (18). This discrepancy may be attributable to between-study differences in exposure levels, methodologies, and/or types of WAGs (19).

In scientific literature, there are several analytical methods for the analysis of volatile anesthetics, such as for the simultaneous determination of SEV and HFIP in plasma samples with headspace-GC–MS (20) or GC–MS/MS (6), and in urine samples using HSSE GC–MS (21) or static headspace-GC–MS (4, 22). An additional study describes the simultaneous analysis of DES, SEV, and HFIP, by using an SPME-GC–MS methodology (23). It exists no method for simultaneous determination of all anesthetics including HFIP. Therefore, there is the need to expand the multi-analytical approach and to validate a sensitive and accurate headspace GC–MS methodology for the simultaneous determination of a greater number of volatile anesthetics in non-invasive samples.

Given the potential risk to some healthcare professionals posed by chronic exposure to WAGs, it is essential to update and improve the corresponding biomonitoring programs. This is one of the objectives of the European Partnership for the Risk of Chemicals (PARC) project (24). The aim of the present study, conceived within the PARC framework, was to develop an analytical method to simultaneously detect and quantify multiple WAG analytes in urine samples from healthcare workers, using headspace gas chromatography coupled to mass spectrometry (HS–GC–MS).

## 2 Materials and methods

### 2.1 Chemicals and reagents

All reagents used were of analytical grade. Analytical standards of the target compounds were DES (99%) (CAS-No. 57041-67-5) from Apollo Scientific (Manchester, United Kingdom), ISO (99%) (CAS-No. 26675-46-7) from TCI Chemicals (Tokyo, Japan), SEV (99%) (CAS-No. 28523-86-6) from Indagoo Research Chemicals (Barcelona, Spain), HFIP (99%) (CAS-No. 920-66-1) from Sigma-Aldrich (Madrid, Spain), and methoxyflurane (MET) (CAS-No. 78-38-0) from Thermo Fisher (Waltham, MA USA). The study also used LC–MS grade methanol (MeOH) from Sigma-Aldrich (Madrid, Spain), sodium chloride (CAS-No. 7647-14-5) from Panreac (Barcelona, Spain), sodium acetate (CAS-No. 631-61-8) from Merck (Darmstadt, Germany), and *β*-glucuronidase from *Helix pomatia* (Sigma-Aldrich). Ultrapure water was obtained with a Milli-Q purification system (Millipore, Bedford, MA, USA).

Working standard solutions for calibration were prepared in MeOH at four concentration levels: 0.1, 1, 10, and 100 mg/L. All solutions were stored at −20 °C in glass containers and used to prepare the calibration standards and quality control samples by stepwise dilution with urine. The internal standard was prepared by dissolving MET in MeOH (2,000 mg/L) followed by serial dilution in MeOH. The final working solution of the internal standard had a concentration of 10 mg/L.

Calibration standards were prepared using spiked urine from a single unexposed individual. Sixteen calibration levels, ranging from 0.1 to 2,000 ng/mL, were prepared by spiking diluted stock solutions into headspace vials containing 5 mL of urine and 0.5 g of sodium chloride. Quantification was performed by adding 10 μL of MET as internal standard.

### 2.2 Urine collection, storage, and sample preparation

In a pilot study, urine samples were collected from healthcare professionals at the San Cecilio University Hospital (HUSC) in Granada (Spain), 37 recently exposed and 24 non-exposed to WAGs (*n* = 61). All exposed participants worked with anesthetics in operating, recovery rooms, obstetrics, or emergency rooms or in any other procedure involving the gases of interest. Non-exposed participants worked in a hospital administration office or outside the hospital (e.g., in the university). All participants gave their written informed consent to participate. DES is the primary anesthetic used at the HUSC, while SEV is mainly used for pediatric and heart surgery. Exposed volunteers were only recruited on Monday or Tuesdays, ensuring an interval of >48 h since entering an operating room and thereby minimizing potential residual concentrations of WAGs. Inclusion criteria for exposed volunteers were their presence in the operating room for at least 2 h prior to urine sample collection, and that the collection of urine sample within 15 min of leaving the room. Urine samples from non-exposed participants were collected at any time during their workday. All urine samples were collected in polypropylene containers. The pilot study was approved by the Provincial Ethics Committee of Granada (# SICEIA-2024-000598).

Samples were prepared by transferring 5 mL of the urine sample to 20 mL headspace vials containing 0.5 g of sodium chloride, performing the transfer immediately after sample collection to prevent analyte loss due to volatility. Additionally, for the analysis of HFIP, the main metabolite of SEV, the vials contained 25 μL of *β*-glucuronidase in 1 M ammonium acetate buffer (pH 5.0). Enzymatic hydrolysis was carried out overnight at 37 °C to ensure the cleavage of HFIP-glucuronide. All urine samples were analyzed, in duplicates, on the day after their collection.

### 2.3 Chromatography and mass spectrometry conditions

Instrumental analysis was performed based on previously published methods (21, 22). Operating conditions of the chromatographic analysis are summarized in Table 1. HS–GC–MS was performed using an Agilent 7890B GC system connected to a CombiPAL RSI 120 Autosampler and an Agilent 7000D triple quadrupole mass spectrometer. Headspace extraction was carried out at an incubation temperature of 65 °C for 10 min under continuous agitation. Following equilibration, 2 mL of headspace vapor was injected into the GC system in split mode (20:1) with the injector temperature set at 220 °C. Helium was used as the carrier gas at a constant flow rate of 1.2 mL/min. A DB-624UI capillary column (60 m × 0.32 mm, 1.8 μm film thickness; 6% cyanopropyl/phenyl, 94% polydimethylsiloxane) was used for separation. The oven was set to start at 30 °C and held for 1 min, then increased by 5 °C/min to 65 °C, followed by a further 15 °C/min increase to 130 °C, when it was held for 2 min. Total analysis time was 15 min. Electron impact (EI) was employed as the ionization mode. Mass selective detection and identification was carried out in Selected-Ion Monitoring (SIM) mode. Data acquisition and processing were performed using MassHunter software version 10.2 SR3.

**Table 1:** Conditions of the headspace gas chromatography method coupled to mass spectrometry (HS–GC–MS).

Headspace conditions	
Injection volume	2 mL
Incubation	10 min (65 °C)
Syringe temperature	65 °C
GC conditions	
Injector	220 °C
Split ratio	20:1
Oven	30 °C (1 min); 5 °C/min, 65 °C (0 min); 15 °C/min, 130 °C (2 min)
Carrier gas	He
Column flow	1.2 mL/min
MS detection	
Ion source	EI (230 °C)
Analysis time	15 min
SIM mode	DES: 101; 51 SEV: 130.9; 180.9; 69 ISO: 117; 149; 51 HFIP: 98.9; 128.9; 79 MET: 81

EI, Electron Ionization; SIM, Selected-Ion Monitoring; DES, Desflurane; SEV, Sevoflurane; ISO, Isoflurane; HFIP, Hexafluoroisopropanol; MET, Methoxyflurane.

### 2.4 Quality assurance

The method was validated according to the analytical guidelines of the European Medicines Agency (25). Linearity and sensitivity were assessed by preparing calibration curves using spiked urine sample from an unexposed individual and analysing the correlation coefficients (*R*^2^). Limit of Detection (LOD) and Limit of Quantification (LOQ) were defined by the lowest analyte concentrations that could be reliably detected and quantified, calculated according to 3·s0 and 10·s0, respectively, where s0 is the standard deviation of the blank. Selectivity was evaluated by analysing blank urine samples to verify the absence of interfering peaks at the retention times of the target analytes.

The accuracy of the method, including its precision and recovery, was evaluated using spiked blank samples at low (6 ng/mL), medium (50 ng/mL), and high (200 ng/mL) concentrations of the selected anesthetic agents (*n* = 6 for each level). Precision was expressed as relative standard deviation (RSD), and accuracy determined through recovery experiments.

Matrix effects were evaluated by comparing the slopes of calibration curves prepared in Milli-Q water and in urine. The assessment was conducted for all analytes within the validated calibration range of 0.1–500 ng/mL. For HFIP, the calibration range was extended to 0.1–1,000 ng/mL due to its wider linear dynamic range and the higher concentrations expected in human biological samples. The percentage matrix effect (ME) was calculated as:


$$\mathrm{ME}\;(\%)=\frac{(\text{Slope of calibration in urine})\;}{\left(\text{Slope of calibration in water}\right)}.100$$

## 3 Results and discussion

### 3.1 Method optimization

Key parameters were first optimized to maximize the analytical efficiency of the method. After testing different headspace incubation times (5, 10, 20, and 30 min), incubation for 10 min yielded the best response. After evaluating incubation temperatures of 55 °C, 65 °C, and 75 °C, 65 °C was the optimal compromise across target analytes. After comparing salt additions of 0.5 g and 1 g per 10 mL sample, the addition of 1 g provided the best reproducibility and highest signal intensity. Finally, the sample volume was reduced to 5 mL, and 0.5 g of salt was added. These conditions were all adopted for the study of urine samples from participants in the pilot study.

### 3.2 GC–MS methodology validation

**Figure 1:**
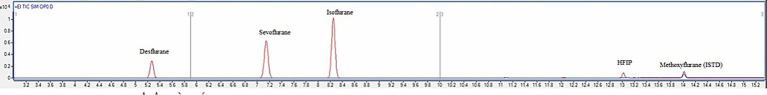
Overlapped chromatograms of a blank and a standard (150 ng/mL) sample in a blank urine sample. Target compounds: Desflurane (DES), Isoflurane (ISO), Sevoflurane (SEV), and its main metabolite, Hexafluoroisopropanol (HFIP).

The method demonstrated satisfactory linearity for the analytes, with *R*^2^ values ranging of 99.5–99.9%. LOD and LOQ values are summarized in Table 2. Selectivity was confirmed, as no interfering peaks were observed at the retention times of the target analytes in blank urine samples. Figure 1 shows a chromatogram of a blank sample, obtained from a non-exposed subject, superimposed on that of a standard concentration sample (150 ng/mL), with adequate peak signaling and no matrix interferences. Accuracy and precision were evaluated at three concentration levels as described in Section 2.4. All relative standard deviation (RSD) values were below 15%, showing adequate intra- and inter-day precision. Recoveries ranged between 85 and 115% for all analytes, fulfilling the established acceptance criteria (Table 3). In addition, ME values of the analytes ranged from 65 to 82%, reflecting the need to use urine from unexposed individuals for matrix calibration. The comparison of the calibration curves obtained is presented in Supplementary Figure S1.

**Table 2:** Parameters of analytical calibration curves.

	*β* (ng/mL)	*R*^2^ (%)	LOD (ng/mL)	LOQ (ng/mL)	LDR (ng/mL)
DES	2.73·10^−2^	99.7	0.30	1.0	0.1–500
SEV	3.65·10^−2^	99.5	0.15	0.5	0.1–500
ISO	3.07·10^−2^	99.5	0.15	0.5	0.1–500
HFIP	2.90·10^−3^	99.9	0.60	2.0	0.5–1,000

*β*, Slope of the calibration curve in matrix; % *R*^2^, Determination coefficient; LOD, Limit of Detection; LOQ, Limit of Quantification; LDR, Linear Dynamic Range; DES, Desflurane; SEV, Sevoflurane; ISO, Isoflurane; HFIP, Hexafluoroisopropanol.

**Table 3:** Recovery assay, precision, and trueness of the method (*n* = 6).

	Spiked (ng/mL)	Measured (ng/mL)	Recovery (%)	RSD (%)
DES	6	6.10	101.65	8.97
	50	49.97	99.94	4.20
	200	99.94	102.40	6.51
ISO	6	6.12	102.04	7.16
	50	48.97	97.94	3.42
	200	190.34	95.17	2.84
SEV	6	5.88	97.98	8.78
	50	48.19	96.37	3.58
	200	166.99	93.39	9.23
HFIP	6	6.55	109.15	7.99
	50	50.16	100.31	10.59
	200	200.04	100.02	6.66

RSD, Relative Standard Deviations; DES, Desflurane; SEV, Sevoflurane; ISO, Isoflurane; HFIP, Hexafluoroisopropanol.

### 3.3 Analysis of samples from healthcare workers exposed to WAGs

Following successful validation of the proposed method, DES, ISO, SEV, and its main metabolite, HFIP, were measured in 37 male and female healthcare workers (anesthesiologists, surgeons, and nurses) who had been exposed to WAGs for at least 2 h (Supplementary Table S1), and in 24 men and women who had not been exposed to anesthetic gases. Each urine sample was analyzed in duplicate and in two different batches (Supplementary Table S1), and the coefficient of variation was calculated to determine the reproducibility of the method.

Urine samples of most of the occupationally exposed healthcare volunteers had detectable concentrations of DES, ranging from <LOQ to 3.80 ng/mL, SEV, ranging from <LOQ to 19.59 ng/mL, and HFIP, ranging from <LOQ to 729.35 ng/mL. ISO, which is not employed as an anesthetic agent at HUSC, was not detected in any urine sample. The most abundant compound was HFIP, the main metabolite of SEV, in line with previous reports (21, 22, 26). In fact, urinary concentrations of DES, SEV, and HFIP were within the range of previous observations, endorsing the use of this biological matrix as a suitable and reliable non-invasive biomarker for assessing occupational exposure to these compounds. Published findings include, for example, reported concentrations of 3.96 ng/mL for SEV (16), 1.64 for SEV and 69.2 ng/mL for HFIP (26), <0.2 ng/mL for SEV and 2.34 ng/mL for HFIP (22). The variability among exposed groups may be attributable to differences in the types of anesthetics and/or in the intensity/duration of the exposure (Table 4). As expected, none of the anesthetic agents examined were detected in the urine samples of the non-exposed volunteers.

**Table 4:** Total urinary concentrations in volunteers (*n* = 61).

	N > LOD	N > LOQ	Mean	P25	P50	P75	Max
Exposed volunteers (*n* = 37)							
DES	10	6	0.53	<LOD	<LOD	0.39	3.80
SEV	27	13	1.39	0.17	0.35	1.39	19.59
HFIP	35	26	49.74	1.95	9.50	28.11	729.35
ISO	–	–	<LOD	<LOD	<LOD	<LOD	<LOD
Non-exposed volunteers (*n* = 24)							
DES	–	–	<LOD	<LOD	<LOD	<LOD	<LOD
SEV	–	–	<LOD	<LOD	<LOD	<LOD	<LOD
HFIP	–	–	<LOD	<LOD	<LOD	<LOD	<LOD
ISO	–	–	<LOD	<LOD	<LOD	<LOD	<LOD

All concentrations are expressed in ng/mL, LOD, Limit of Detection; LOQ, Limit of Quantification; DES, Desflurane; SEV, Sevoflurane; HFIP, Hexafluoroisopropanol; ISO, Isoflurane. ISO was <LOD in all urine samples.

Our results confirm that inhaled anesthetic gases can be absorbed and subsequently excreted through urine. Repeated occupational exposure to anesthetic gases may pose potential health risks and should therefore be monitored. In March 2025, the National Institute for Occupational Safety and Health in Spain updated occupational exposure limits for different chemical agents (27). Neither SEV nor DES were included, but an Environmental Limit Value for Daily Exposure (VLA-ED®) of 50 ppm was declared for ISO. Other European countries have established Time-Weighted Average (TWA) limits ranging from 5 to 10 ppm for DES and below 10 ppm for SEV and ISO (28–31).

The simultaneous detection of multiple fluorinated anesthetics in urine supports the usefulness of this biological matrix as a non-invasive biomarker of occupational exposure to these compounds. The results of the double determination of the samples further validate the analytical method used and confirm its robustness for detecting trace levels under real-world working conditions. Continuous biomonitoring is crucial to elucidate internal dose dynamics, to assess potential bioaccumulation, and to evaluate the long-term health impact on exposed healthcare professionals. An occupational study is under way applying the same methodology to determine the effects of exposure to these WAGs on anesthesiologists, surgeons, and other professionals in the operating room.

## 4 Conclusion

In this study, a multianalyte HS–GC–MS method for analysing anesthetic gases in human urine samples was optimized and validated. It permits the simultaneous determination of multiple WAGs. To our best knowledge, the four compounds under study have not previously been analyzed in a simultaneous manner. The method was validated in full accordance with international analytic validation guidelines and applied to 61 urine samples from exposed and non-exposed workers. The results and comparison with previous studies support the validity of the developed analytical method, demonstrating adequate sensitivity and reproducibility under real working conditions. Application of this methodology in occupational studies can serve to review exposure limits for the most widely used anesthetic agents.

## Data Availability

The original contributions presented in the study are included in the article/Supplementary material, further inquiries can be directed to the corresponding author.
