# Supplementary material for: A multianalyte method for simultaneous evaluation of occupational exposure to anesthetic gases in urine samples from healthcare professionals
Source: Front Public Health. 2026 Apr 16;14:1802993. doi: 10.3389/fpubh.2026.1802993 (PMC13130493; doi:10.3389/fpubh.2026.1802993)
Supplement: Supplementary file 1 [file Supplementary_File_1.docx]

Supplementary Material

| **Figure S1.** Calibration curves in urine and water matrix. | |
| --- | --- |
| 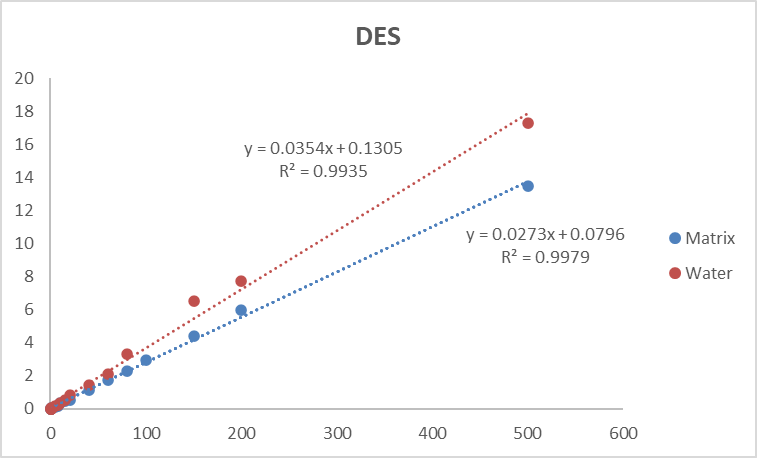 | 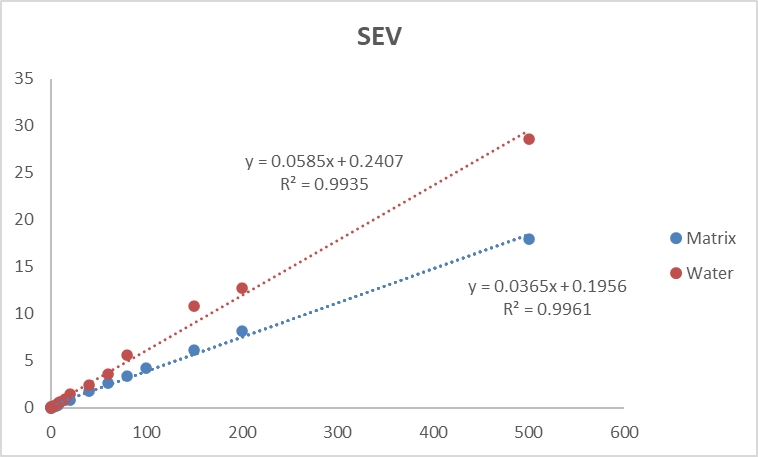 |
| 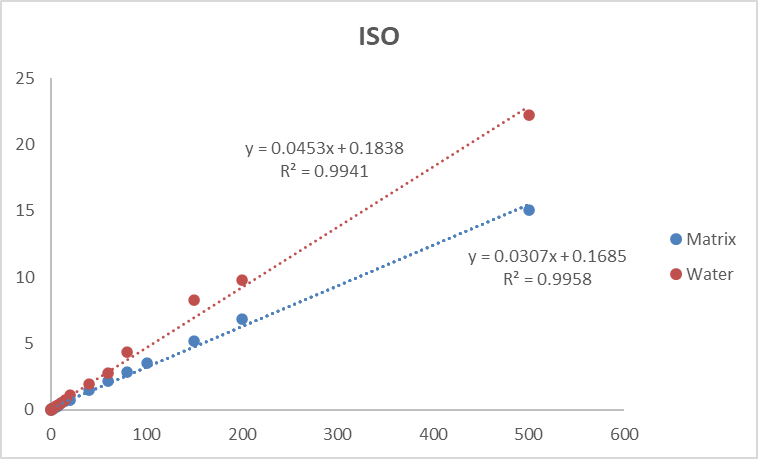 | 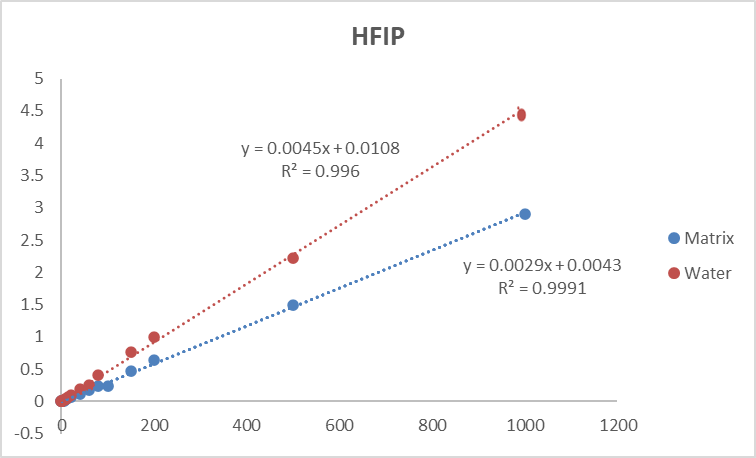 |
| DES: Desflurane; SEV: Sevoflurane; ISO: Isoflurane; HFIP: Hexafluoroisopropanol | |

| **Table S1.** Urinary anesthetics concentrations found in samples of volunteers. | | | | |
| --- | --- | --- | --- | --- |
| **Sample** | **DES** | **SEV** | **HFIP** | **ISO** |
| **Exposed (n=37)** | | | | |
| **S01** | ND | D | 2.21 (18.96) | ND |
| **S02** | 1.25 (9.60) | ND | ND | ND |
| **S03** | 1.30 (5.92) | ND | ND | ND |
| **S04** | 1.02 (9.00) | 0.92 (1.28) | 45.70 (8.11) | ND |
| **S06** | ND | ND | 40.67 (3.48) | ND |
| **S07** | ND | 4.19 (7.56) | 587.94 (2.64) | ND |
| **S08** | 1.75 (1.48) | ND | ND | ND |
| **S09** | 1.84 (6.97) | ND | ND | ND |
| **S10** | ND | 1.23 (9.65) | 11.05 (8.01) | ND |
| **S11** | ND | 1.72 (2.25) | 34.84 (3.19) | ND |
| **S12** | ND | D | 7.55 (7.05) | ND |
| **S13** | D | 0.52 (10.71) | 13.96 (6.16) | ND |
| **S16** | D | D | 7.25 (1.98) | ND |
| **S18** | ND | D | 10.06 (18.65) | ND |
| **S22** | ND | 19.59 (3.82) | 729.35 (11.86) | ND |
| **S23** | ND | 1.49 (6.53) | 26.02 (1.34) | ND |
| **S24** | ND | 1.53 (5.40) | 32.44 (2.66) | ND |
| **S25** | ND | 0.83 (8.12) | 28.11 (7.25) | ND |
| **S27** | ND | D | 9.50 (6.96) | ND |
| **S28** | ND | D | D | ND |
| **S29** | 3.80 (1.46) | ND | ND | ND |
| **S30** | ND | D | 3.03 (5.10) | ND |
| **S32** | D | D | D | ND |
| **S40** | ND | 4.86 (14.32) | 27.01 (8.37) | ND |
| **S44** | ND | 2.40 (2.43) | 55.87 (1.13) | ND |
| **S45** | ND | 6.66 (3.33) | 37.29 (2.31) | ND |
| **S51** | ND | ND | 10.26 (19.43) | ND |
| **S52** | ND | D | 12.98 (3.71) | ND |
| **S53** | ND | D | 7.28 (1.96) | ND |
| **S54** | ND | ND | 2.28 (13.00) | ND |
| **S55** | ND | D | 80.02 (0.39) | ND |
| **S56** | ND | D | D | ND |
| **S57** | ND | ND | 8.45 (12.02) | ND |
| **S58** | ND | ND | D | ND |
| **S59** | ND | D | D | ND |
| **S60** | D | D | 3.49 (12.12) | ND |
| **S61** | ND | D | D | ND |
| **Non-exposed (n=24)** | | | | |
| **S05** | ND | ND | ND | ND |
| **S14** | ND | ND | ND | ND |
| **S15** | ND | ND | ND | ND |
| **S17** | ND | ND | ND | ND |
| **S19** | ND | ND | ND | ND |
| **S20** | ND | ND | ND | ND |
| **S21** | ND | ND | ND | ND |
| **S26** | ND | ND | ND | ND |
| **S31** | ND | ND | ND | ND |
| **S33** | ND | ND | ND | ND |
| **S34** | ND | ND | ND | ND |
| **S35** | ND | ND | ND | ND |
| **S36** | ND | ND | ND | ND |
| **S37** | ND | ND | ND | ND |
| **S38** | ND | ND | ND | ND |
| **S39** | ND | ND | ND | ND |
| **S41** | ND | ND | ND | ND |
| **S42** | ND | ND | ND | ND |
| **S43** | ND | ND | ND | ND |
| **S46** | ND | ND | ND | ND |
| **S47** | ND | ND | ND | ND |
| **S48** | ND | ND | ND | ND |
| **S49** | ND | ND | ND | ND |
| **S50** | ND | ND | ND | ND |
| All concentrations are expressed in ng/mL [mean CV(%)], CV: Coefficient of variation; ND: Not detected (<LOD); D: Detected (>LOD and <LOQ), DES: Desflurane, SEV: Sevoflurane, HFIP: Hexafluoroisopropanol, ISO: Isoflurane. All samples for ISO were <LOD. | | | | |
